# *In Vitro* Gene Delivery Mediated by Asialofetuin-Appended Cationic Liposomes Associated with **γ**-Cyclodextrin into Hepatocytes

**DOI:** 10.1155/2011/476137

**Published:** 2010-12-09

**Authors:** Keiichi Motoyama, Yoshihiro Nakashima, Yukihiko Aramaki, Fumitoshi Hirayama, Kaneto Uekama, Hidetoshi Arima

**Affiliations:** ^1^Graduate School of Pharmaceutical Sciences, Kumamoto University, 5-1 Oe-honmachi, Kumamoto 862-0973, Japan; ^2^School of Pharmacy, Tokyo University of Pharmacy and Life Sciences, 1432-1 Horinouchi, Hachioji, Tokyo 192-0392, Japan; ^3^Faculty of Pharmaceutical Sciences, Sojo University, 4-22-1 Ikeda, Kumamoto 860-0082, Japan

## Abstract

The purpose of this study is to evaluate *in vitro* gene delivery mediated by asialofetuin-appended cationic liposomes (AF-liposomes) associating cyclodextrins (CyD/AF-liposomes) as a hepatocyte-selective nonviral vector. Of various CyDs, AF-liposomes associated with plasmid DNA (pDNA) and **γ**-cyclodextrin (**γ**-CyD) (pDNA/**γ**-CyD/AF-liposomes) showed the highest gene transfer activity in HepG2 cells without any significant cytotoxicity. In addition, **γ**-CyD enhanced the encapsulation ratio of pDNA with AF-liposomes, and also increased gene transfer activity as the entrapment ratio of pDNA into AF-liposomes was increased. **γ**-CyD stabilized the liposomal membrane of AF-liposomes and inhibited the release of calcein from AF-liposomes. The stabilizing effect of **γ**-CyD may be, at least in part, involved in the enhancing gene transfer activity of pDNA/**γ**-CyD/AF-liposomes. Therefore, these results suggest the potential use of **γ**-CyD for an enhancer of transfection efficiency of AF-liposomes.

## 1. Introduction

The principle of somatic gene therapy is that genes can be introduced into selected cells in the body in order to treat genetic or acquired diseases. The liver may be potentially an important target for gene therapy, because crucial diseases such as amyloidosis, primary biliary cirrhosis, familial hypercholesteremia, phenyl ketonuria, and virus hepatitis occur in this organ [1]. In addition, the liver has the ability to synthesize a wide variety of proteins, to perform various posttranslational modifications, and to secrete them into the blood.

Of various nonviral methods, the lipofection method, by which cationic lipids (cationic liposomes) are used for transfection and interact with plasmid DNA (pDNA) to give a lipoplex, has recently attracted attention [2]. Cationic liposomes have great advantages as gene delivery carriers such as (1) low cytotoxicity and immunogenicity [3], (2) regulation of the pharmacokinetics through the modification of particle size or lipids components of liposomes [4], (3) entrapment of pDNA into inner water phase of liposomes and suppression of DNA degradation by DNase [5], and (4) delivery of gene to target cells by the addition of target ligands and/or antibody [6]. 

Asialofetuin (AF) is a glycoprotein that possesses three asparagine-linked triantennary complex carbohydrate chains with terminal *N*-acetylgalactosamine residues. The protein displays affinity to asialoglycoprotein receptor (ASGP-R) on hepatocytes and enters the cells through the receptor [7, 8]. Thus, AF has been used as a ligand to deliver drugs to hepatocytes and a competitive inhibitor to ASGP-R [9, 10]. In fact, the widespread use of AF-appended liposomes (AF-liposomes) as a hepatocyte-selective gene transfer carrier has been reported [11, 12]. 

Cyclodextrins (CyDs) have recently been applied to gene transfer and oligonucleotide delivery [13–16]. CyDs are cyclic (*α*-1,4)-linked oligosaccharides of *α*-D-glucopyranose containing a hydrophobic central cavity and hydrophilic outer surface, and they are known to be able to act as novel host molecules by chemical modification [17]. Davis and his colleagues reported that the ternary complex of a water-soluble *β*-CyD polymer with 6^A^,6^D^-dideoxy-6^A^,- 6^D^-di-(2-aminoethanethio)-*β*-CyD and dimethylsuberimidate (*β*CDP6), galactosylated, or transferrin polyethylene glycol conjugates with adamantane, and pDNA possesses higher transfection efficiency in hepatoma or leukemia cells, respectively, through receptor-mediated endocytosis [18, 19]. Recently, we reported the potential use of PAMAM dendrimer functionalized with *α*-CyD (*α*-CDE) [20] and lactosylated *α*-CDE (Lac-*α*-CDE) as a hepatocyte specific gene delivery *in vitro* and *in vivo *[21]. Meanwhile, Lawrencia et al. reported that lipoplex transfection of pDNA with DOTAP (*N*-[1-(2,3-dioleoyloxy)propyl]-*N*,*N*,*N*-trimethylammonium methyl-sulfate) in the presence of cholesterol, which is solubilized by methyl-*β*-cyclodextrin (methyl-*β*-CyD), has significantly improved transfection efficiency in urothelial cells due to change in membrane fluidity by methyl-*β*-CyD [22]. In addition, we previously demonstrated that intravenous injection of the pegylated liposomes entrapping the doxorubicin (DOX) complex with *γ*-CyD in BALB/c mice bearing Colon-26 tumor cells showed DOX accumulation in tumor tissues and the potent antitumor effect, compared with those of DOX solution and pegylated liposomes entrapping DOX alone [23]. These lines of evidence suggest that transfection efficiency and pharmacokinetics of pDNA can be altered by the association of CyDs with AF-liposomes. 

Based on these backgrounds, the purpose of this study is to evaluate *in vitro* gene delivery of AF-liposomes associated with CyDs as a hepatocyte-selective nonviral vector in HepG2 cells. In addition, the mechanisms by which *γ*-CyD enhanced transfection efficiency of pDNA/AF-liposomes were investigated in the view of a receptor recognition, physicochemical properties (particle size, *ζ*-potential, and encapsulation ratio), membrane fluidity, cellular uptake, and cytotoxicity of AF-liposomes.

## 2. Materials and Methods

### 2.1. Materials

Dilauroylphosphatidylcholine (DLPC), dioleoylphosphatidylethanolamine (DOPE), dipalmitoylphosphatidylethanolamine (DPPE), and diacylphosphatidylethanolamine-N-lissamine rhodamine B sulfonyl (RH-PE) were obtained from Avanti Polar-Lipid (Alabama). *N*-(*α*-Trimethylammonioacetyl)-didodecyl-D-glutamate chloride (TMAG) was purchased from Sogo Pharmaceutical (Tokyo, Japan). Asialofetuin (AF) and 2-mercaptoethanol were obtained from Sigma Chemical (St. Louis, MO). *N*-hydroxysulfosuccinimide (Sulfo-NHS) was purchased from Fluka (Buchs, Switzerland). 1-Ethyl-3-(3-dimethylaminopropyl) carbodiimide (EDC) was from Dojindo (Kumamoto, Japan). 2-(*N*-morpholino) ethanesulfonic acid (MES) and 2-[4-(2-hydroxyethyl)-1-piperazinyl] ethanesulfonic acid (HEPES) were purchased from Nacalai Tesque (Kyoto, Japan). CyDs used in this study were supplied by Nihon Shokuhin Kako (Tokyo, Japan) (Table 1). The average degrees of substitution of 2-hydroxypropyl group in 2-hydroxypropyl-*α*-CyD (HP-*α*-CyD), HP-*β*-CyD, and HP-*γ*-CyD are 4.0, 4.8 and 4.3, respectively. Fetal calf serum (FCS) was obtained from Nichirei (Tokyo, Japan). Dulbecco's modified Eagle's medium (DMEM) was purchased from Nissui Pharmaceuticals (Tokyo, Japan). Plasmid pRL-CMV-Luc vector encoding Renilla luciferase and having a CMV promoter as well as pGL3-control vector encoding firefly luciferase and having a SV40 promoter were obtained from Promega (Tokyo, Japan). pEGFP N1 DNA encoding EGFP and having a CMV promoter was purchased from BD Bioscience Clontech (San Jose, CA). These DNA vectors were abbreviated to pDNA. The purification of pDNA amplified in bacteria was carried out using QIAGEN EndoFree plasmid MAXI kit (<0.1 EU/*μ*g endotoxin). Picogreen dsDNA reagent and ULYSIS Alexa Fluor 488 (Alexa) Nucleic Acid Labeling Kit were purchased from Molecular Probes (Tokyo, Japan). Bovine serum albumin (BSA) was obtained from Roche Diagnostics (Tokyo, Japan). Other chemicals and solvents were of analytical reagent grade, and deionized double-distilled water was used throughout the study.

### 2.2. Preparation of AF-Liposomes

**Figure 1:**
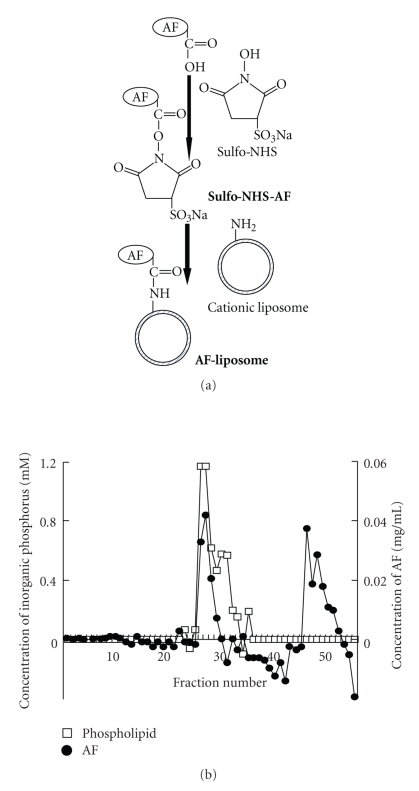
Preparation of asialofetuin-modified liposomes (AF-liposomes). (a) Preparation pathway of AF-liposomes. (b) Elution profiles of AF-liposomes determined by gel filtration chromatography.

Preparation of AF-liposomes was performed according to the method reported by Hara et al. [11] with some modifications (Figure 1). Briefly, lipids mixtures, DLPC/TMAG/DOPE/DPPE (3/2/4/1, molar ratio, the amount of total lipids was 30 *μ*mol), were dissolved in chloroform, and the solvent was removed under reduced pressure by a rotary evaporator. After addition of 5 mL of 0.1 M MES buffer (activation buffer, pH 5.0) containing 0.9% NaCl, the solution was sonicated by a bath type of sonicator (ultrasonic automatic washer US-4, AS ONE, Osaka, Japan) for 5 min under gassing with nitrogen, and then small unilamellar vesicle, large unilamellar vesicle, and/or small oligolamellar liposomes were obtained. Next, 6 mg of AF was dissolved in 1 mL of activation buffer, which contained 3.3 mg of Sulfo-NHS and 1.2 mg of EDC. After mixing for 15 min at room temperature, the reaction was stopped by the addition of 4.2 *μ*L of 2-mercaptothanol and obtained Sulfo-NHS-AF. Sulfo-NHS-AF was mixed with LUV and stirred for 2 h at room temperature. After addition of hydroxylamine HCl, AF-liposomes were obtained. To remove the free AF, we performed gel filtration using Sepharose CL-4B column (Amersham Pharmacia Biotech, Freiburg, Germany) and determined phospholipids and AF by the Bartlett method [24] and the Bradford method [25], respectively. The fractions number 26–30, which eluted both phospholipids and AF, were collected as the AF-liposomes fraction (Figure 1(b)). The *ζ*-potential value and particle size of AF-liposomes were 31.2 ± 0.1 mV and 230.7 ± 4.2 nm, respectively. The amount of AF modification in 1 *μ*mol of AF-liposome lipids was 35.8 *μ*g/*μ*mol lipids, indicating that AF modification rate against DPPE in liposomes was 0.75%. Liposomes without AF modification (N-liposomes) was prepared by the solution without Sulfo-NHS-AF, and the other procedure was the same as that of AF-liposomes.

### 2.3. Preparation of pDNA/CyDs/AF-Liposomes

Preparation of pDNA/CyD/AF-liposomes was performed according to the method reported by Hara et al. with some modifications [12]. Briefly, 2 *μ*L of the solution containing pDNA (1 *μ*g/*μ*L) and CyDs (1 *μ*M/*μ*M lipids) dissolved in TE buffer were added to AF- or N-liposomes suspension. After mixing, the solution was freeze-dried. Then, the sample was rehydrated with THBS (10 mM, pH 7.5) for 30 min. After freezing-thawing for three times, the vesicles were extruded through PVDF membranes (Nucleopore, Plesanton, CA) with pores of diameter 450 and 200 nm. The filtrates were used for further experiments as pDNA/CyDs/AF-liposomes or pDNA/CyDs/N-liposomes. The entrapment ratios of CyDs were evaluated by the anthrone-sulfuric acid method [26]. Briefly, 3 mL of anthrone reagent was added to 0.5 mL of the suspension containing CyDs/liposomes. The tube was covered with a glass ball and was heated for 10 min in boiling water. After quenching with cold water, absorbance of the suspension was measured by a U-2000A spectrophotometer (Hitachi, Tokyo, Japan) at 620 nm. The encapsulation ratios of pDNA were determined by a fluorescent spectrometer F-4500 (Hitachi, Tokyo, Japan). Briefly, 350 *μ*L of the suspension containing pDNA/CyD/AF-liposomes in 10 mM THBS (pH 7.5) were mixed with 200 times diluted Picogreen dsDNA reagent (350 *μ*L). After incubation for 30 min at 25°C, fluorescent intensity (*F*_*p*_*o*__) was determined. Next, after addition of 20% of Triton-X (20 *μ*L) to the sample, fluorescent intensity (*F*_*p*_*t*__) was determined and the encapsulation ratio of pDNA was calculated as follows: encapsulation ratio (%) = [(*F*_*p*_*t*__ · *r* − *F*_*p*_*o*__)/*F*_*p*_*t*__ · *r*] · 100, where *r* is compensation coefficient (*r* = 1.03). Particle size and *ζ*-potential value of liposomes in 10 mM THBS (pH 7.5) were measured by a submicron particle analyzer N4 Plus (Beckman Coulter, Fullerton, CA) and ELS-8000 (Otsuka Electronics, Osaka, Japan), respectively.

### 2.4. Interaction of CyDs with AF-Liposomes

AF-liposomes encapsulating calcein were prepared by the freezing and thawing method after addition of 0.1 mM calcein in 10 mM THBS (pH 7.5). The vesicles were extruded through two stacked polycarbonate membranes (Nucleopore, Plesanton, CA) with pores of diameter 1 *μ*m. The sample was subjected to 10 passes through the filter at 40°C. The filtrates were extruded through the polycarbonate membranes (pore size 0.2 *μ*m) as described above. Two milliliters of CyDs solution adjusted at the appropriate concentration (5–20 mM) using 10 mM phosphate buffer were added to 20 *μ*L of the liposomal suspension, and then the resulting suspension was incubated for 30 min at 25°C. The fluorescence intensity of calcein (*F*_*t*_) was measured with a fluorophotometer (Hitachi F-4500, Tokyo, Japan) at 25°C; excitation and emission wavelengths were 490 and 520 nm, respectively. After addition of 20 *μ*L of cobalt chloride solution (10 mM) to the sample to quench the fluorescence of nonencapsulated calcein, the intensity of fluorescence of encapsulated calcein (*F*_*in*_) was also determined. Then, the liposomes were completely disrupted by the addition of 20 *μ*L of Triton X-100 (20%) solution, and the intensities of fluorescence after quenching by cobalt chloride (*F*_*q*_) were measured. Calcein encapsulation ratio was calculated by the equation as follows: encapsulation ratio (%) = [(*F*_*in*_ − *F*_*q*_ · *r*)/(*F*_*t*_ − *F*_*q*_ · *r*)] · 100, where *r* is compensation coefficient (*r* = 1.04).

### 2.5. Cell Culture

HepG2 cells, a human hepatocellular carcinoma cell line, A549 cells, a adenocarcinomic human alveolar basal epithelial cells, and NIH3T3 cells, a mouse embryonic fibroblast cell line, were obtained from Riken Bioresource Center (Tsukuba, Japan). HepG2, A549, and NIH3T3 cells were grown in DMEM, containing 1 × 10^5^ U/L of penicillin, 0.1 g/L of streptomycin supplemented with 10% FCS at 37°C in a humidified 5% CO_2_ and 95% air atmosphere.

### 2.6. In Vitro Gene Transfer

*In vitro *transfection of the pDNA/CyDs/AF-liposomes was performed utilizing the luciferase expression of pDNA (pRL-CMV-Luc or pGL3-control vector) in HepG2, A549, and NIH3T3 cells. The cells (2 × 10^5^ cells per 24 well plate) were seeded 6 h before transfection and then washed twice with 500 *μ*L of serum-free medium. Two hundred *μ*L of serum-free medium containing pDNA/CyDs/AF-liposomes in the absence and presence of AF as a competitor protein or BSA as a control protein were added to each dish and then incubated at 37°C for 3 h. After washing HepG2 cells with serum-free medium twice, 500 *μ*L of medium containing 10% FCS were added to each dish and then incubated at 37°C for 21 h. After transfection, the gene expression was measured as follows: Renilla and firefly luciferase contents in the cell lysate were quantified by a luminometer (Lumat LB9506, EG&G Berthold Japan, Tokyo, Japan) using the Promega Renilla and firefly luciferase assay reagent (Tokyo, Japan), respectively. It was confirmed that CyDs and AF-liposomes had no influence on the luciferase assays under the present experimental conditions. Total protein content of the supernatant was determined by Bio-Rad protein assay kit (Bio-Rad Laboratories, Tokyo, Japan). EGFP-expressing cells were determined by a confocal laser scanning microscopy (CLSM, Olympus FV300-BXCarl Zeiss LSM-410, Tokyo, Japan) with an argon laser at 488 nm after fixation. Briefly, the cells (2 × 10^5^ cells per 35 mm glass bottom dish) were seeded 6 h before transfection and then washed twice with 500 *μ*L of serum-free medium. Transfection with pEGFP N1 DNA was performed using the same protocol as described above. The EGFP expression ratio was determined by the number of EGFP-expressing cells per 100 cells. To observe the cellular uptake of Rhodamine-labeled AF-liposomes (RH-AF-liposomes) and Alexa-labeled pDNA (Alexa-pDNA), HepG2 cells (2 × 10^5^ cells/dish) were incubated with the Alexa-pDNA/CyDs/RH-AF-liposomes for 3 h. After incubation, the cells were rinsed with PBS (pH 7.4) twice and fixed in methanol at 4°C for 5 min prior to observation by a CLSM.

### 2.7. Reverse Transcriptase-Polymerase Chain Reaction (RT-PCR)

Total RNA was isolated using an RNeasy Mini Kit (Qiagen, Tokyo, Japan) according to manufacturer's procedure. The synthesis of the first-strand cDNA was carried out with SuperScript III reverse transcriptase (Invitrogen, Carlsbad, CA). Approximately, 1 *μ*M random primer was annealed to 3 *μ*g of total RNA and extended with 1 *μ*L of reverse transcriptase in 10 *μ*L of reaction containing 4 *μ*L of 5 × first-strand buffer, 1 *μ*L of deoxyribonucleotide triphosphate (dNTPs), and 1 *μ*L of dithiothreitol. Reverse transcription was carried out at 42°C for 50 min. The expression of mRNA transcripts of *Renilla luciferase* (forward: 5′-GGCTGACCGCCCAACGACCCCC-3′, reverse: 5′-GACGTCAATAGGGGGCGGACTTGG-3′) and human *β-actin* (forward: 5′- TCCTGTGGCATCCATCCACGAAACT-3′, reverse: 5′-GAAGCATTTGCGGTGGACGAT-3′) was determined by RT-PCR. PCR amplification was carried out in a PCR Thermal Cycler (Takara Bio, Shiga, Japan). PCR was conducted in a total volume of 100 *μ*L with 2 *μ*L of the cDNA solution, 2 *μ*L of each 10 mM dNTP, 2.5 U of TaKaRa Ex *Taq* DNA polymerase, and 500 nM of both forward and reverse primers. The thermal cycling conditions were set to 95°C for 11 min, followed by 20 cycles of amplification at 94°C for 1 min, 55°C for 1 min, and 72°C for 2 min for denaturing, annealing, and extension. After the last cycle, the samples were incubated at 72°C for 7 min. The amplified products were separated on 2% agarose gels by electrophoresis and visualized with 0.1% ethidium bromide under UV light.

### 2.8. Cytotoxicity

The effects of pDNA/CyDs/AF-liposomes on cell viability were measured as reported previously [27]. The transfection was performed as described in the transfection section. After washing twice with Hanks' balanced salt solutions (HBSS, pH 7.4) to remove pDNA and/or AF-liposomes, 270 *μ*L of fresh HBSS and 30 *μ*L of  WST-1 reagent were added to the plates and incubated at 37°C for 30 min. The absorbance of the solution was measured at 450 nm, with referring absorbance at 655 nm, with a Bio-Rad Model 550 microplate reader (Bio-Rad Laboratories, Tokyo, Japan).

### 2.9. Membrane Fluidity of Liposomes

To evaluate the thermodynamic characterization of liposomes in the presence of CyDs, differential scanning calorimetry (DSC) measurements were performed. Briefly, N-liposome or DLPC-liposome suspension (5 *μ*mol/mL of lipids) and CyDs (10–50 mM) solution were mixed, and the solution was analyzed by a Microcal MC2 scanning calorimeter (Northampton, MA) with a scanning rate of 1°C/min in the range of 4–70°C. From the thermographs, membrane phase-inversion temperature and its enthalpy (ΔHcal) were calculated.

### 2.10. Statistical Analysis

Data are given as the mean  ±  SEM. Statistical significance of means for the studies was determined by analysis of variance followed by Scheffe's test. *P* values for significance were set at  .05.

## 3. Results

### 3.1. Interaction between AF-Liposomes and CyDs

**Figure 2:**
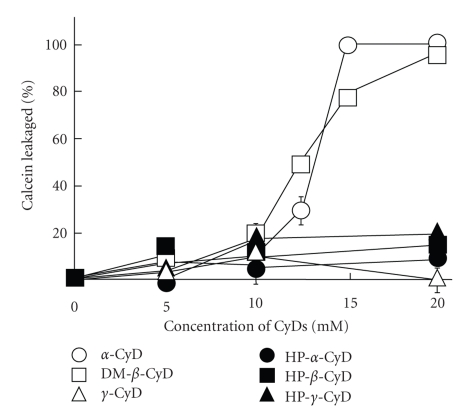
Effects of CyDs on leakage of calcein from calcein-encapsulated AF-liposomes in isotonic Tris-HCl-buffered saline (10 mM, pH 7.5) at 25°C. Calcein-encapsulated AF-liposomes were incubated with various CyDs for 30 min, and the concentrations of calcein released from AF-liposomes were determined using a fluorospectrophotometer. Each point represents the mean  ±  SEM of 3 experiments.

CyDs have been reported to interact with cell membrane constituents such as cholesterol and phospholipids, resulting in the induction of hemolysis of human and rabbit red blood cells at high concentrations of CyDs [28–30]. In addition, CyDs are well known to disrupt liposomal membranes, depending on CyD cavity sizes and membrane components [31, 32]. Then, we evaluated the interaction of CyDs with AF-liposomes by measuring the leakage of calcein, a fluorescent marker, from calcein-encapsulated AF-liposomes in isotonic Tris-HCl-buffered saline (pH 7.5) (Figure 2). *α*-CyD and DM-*β*-CyD significantly increased calcein leakage from calcein-encapsulated AF-liposomes after incubation for 30 min in a concentration-dependent manner. On the other hand, calcein leakage in the presence of *γ*-CyD and three types of HP-CyDs was low even at the concentration of 20 mM CyDs. These results suggest that the interaction of *γ*-CyD and HP-CyDs with AF-liposomes was weaker than that of *α*-CyD and DM-*β*-CyD.

### 3.2. In Vitro Gene Delivery of AF-Liposomes Associated with CyDs

**Figure 3:**
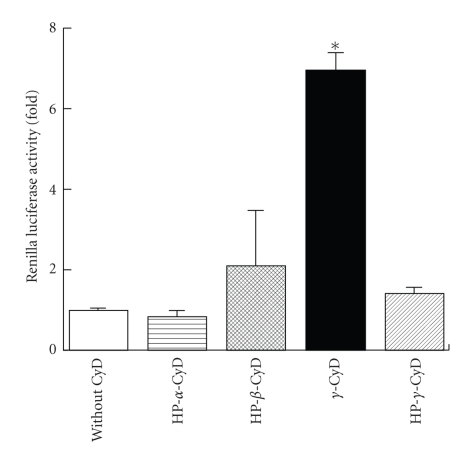
Gene transfer activity of pDNA/CyDs/AF-liposomes in HepG2 cells. pDNA was pRL-CMV. The charge ratio of AF-liposomes/pDNA was 1.6. CyDs were added to AF-liposomes suspension before freeze-drying. The concentration of CyD was 1 *μ*M/*μ*M lipids. Cells were incubated with pDNA/*γ*-CyDs/AF-liposomes for 3 h in FCS-free medium. After washing twice, the cells were incubated for 21 h in culture medium supplemented with 10% FCS. The luciferase activity in cell lysates was determined using a luminometer. Each value represents the mean  ±  SEM of 3–7 experiments. *P < .05  versus without CyD.

**Figure 4:**
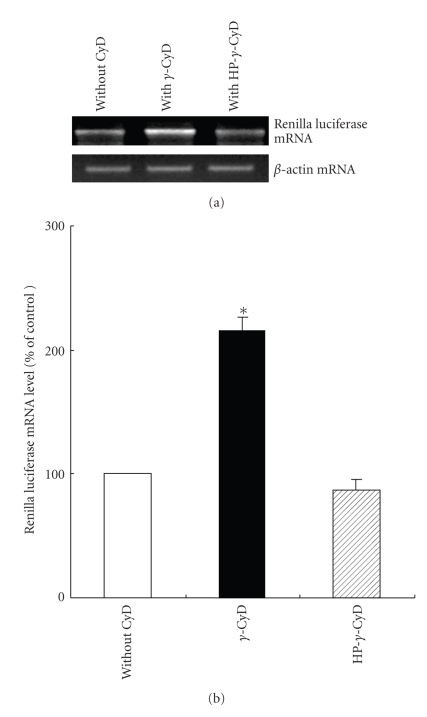
Effect of *γ*-CyDs on *Renilla luciferase* mRNA levels after transfection of pDNA/AF-liposomes or pDNA/*γ*-CyDs/AF-liposomes in HepG2 cells. The charge ratio of AF-liposomes/pDNA was 1.6. CyDs were added to AF-liposomes suspension before freeze-drying. The concentration of CyD was 1 *μ*M/*μ*M lipids. Cells were incubated with pDNA/*γ*-CyDs/AF-liposomes for 3 h in FCS-free medium. After washing twice, the cells were incubated for 21 h in culture medium supplemented with 10% FCS. The *Renilla luciferase *mRNA level in HepG2 cells was assayed by RT-PCR. The *luciferase* mRNA level in each sample was normalized to abundance of *β-actin* mRNA. Each value represents the mean  ±  SEM of 3–7 experiments. *P < .05  versus without CyD.

Next, we evaluated *in vitro* gene transfer activity of pDNA/CyDs/AF-liposomes in HepG2 cells, ASGP-R positive cells (Figure 3). Here, we used pRL-CMV (CMV promoter) as pDNA and the charge ratio (AF-liposomes/pDNA) of 1.6 optimized by our previous study (data not shown). The gene transfer activity of pDNA/*γ*-CyD/AF-liposomes in HepG2 cells was significantly higher than that of pDNA/HP-*α*-, HP-*β*-, and HP-*γ*-CyDs/AF-liposomes (Figure 3). Next, we measured the *Renilla luciferase* mRNA level after transfection of pDNA/CyDs/AF-liposomes in HepG2 cells by the RT-PCR method (Figure 4). As predicted, *γ*-CyD significantly increased the luciferase expression, but HP-*γ*-CyD did not, suggesting that *γ*-CyD is involved in the enhancing effect on luciferase expression at or prior to a transcription process. 

**Figure 5:**
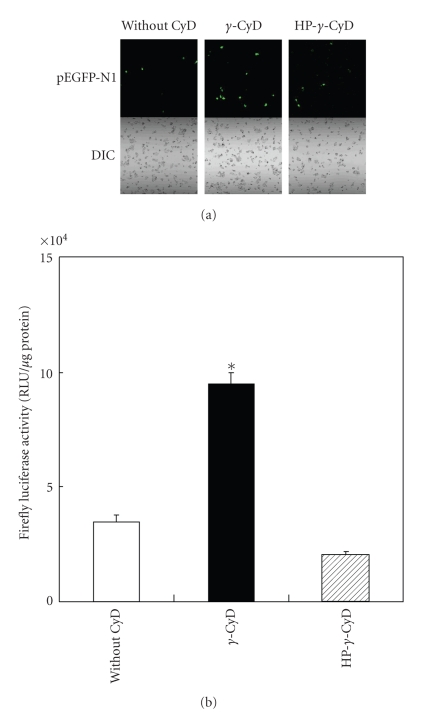
Gene transfer efficiency of pDNA/AF-liposomes or pDNA/*γ*-CyDs/AF-liposomes in HepG2 cells. pDNA was (a) pEGFP-N1 or (b) pGL3-control vector. The charge ratio of AF-liposomes/pDNA was 1.6. *γ*-CyDs were added to AF-liposomes suspension before freeze-drying. The concentration of CyDs was 1 *μ*M/*μ*M lipids. Cells were incubated with pDNA/*γ*-CyDs/AF-liposomes for 3 h in FCS-free medium. After washing twice, the cells were incubated for 21 h in culture medium supplemented with 10% FCS. (a) Cells were determined by a confocal laser scanning microscopy. The percentage in parenthesis represents frequency rate of EGFP expression cells. (b) The luciferase activity in cell lysates was determined using a luminometer. Each value represents the mean  ±  SEM of 3–7 experiments. *P < .05  versus without CyD.

**Figure 6:**
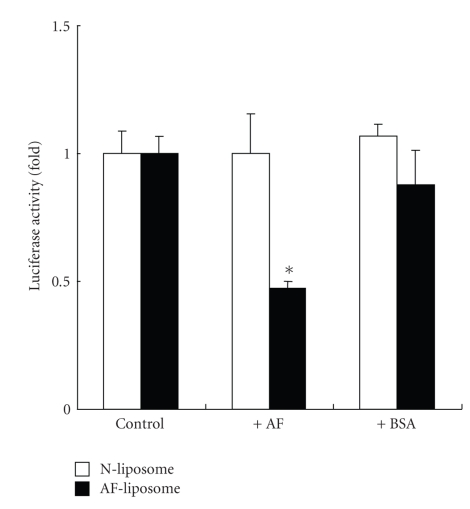
Effects of competitors on gene transfer activity of pDNA/*γ*-CyD/N-liposomes and AF-liposomes in HepG2 cells. pDNA was pRL-CMV. The charge ratio of AF-liposomes/pDNA was 1.6. *γ*-CyD was added to AF-liposomes suspension before freeze-drying. The concentration of *γ*-CyD was 1 *μ*M/*μ*M lipids. Cells were incubated with pDNA/*γ*-CyDs/AF-liposomes for 3 h in FCS-free medium. After washing twice, the cells were incubated for 21 h in culture medium supplemented with 10% FCS. The luciferase activity in cell lysates was determined using a luminometer. The concentrations of AF and BSA were 5 mg/mL. Each value represents the mean ± SEM of 3–7 experiments. *P < .05  versus control.

**Figure 7:**
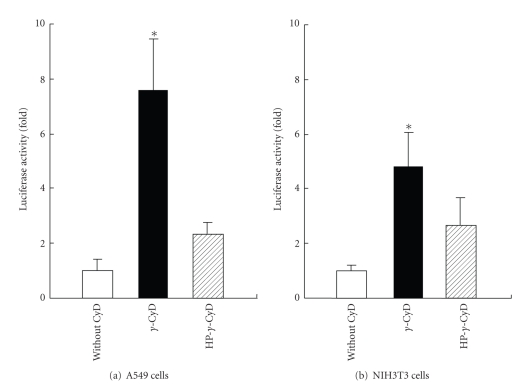
Gene transfer activity of pDNA/CyDs/AF-liposomes in (a) A549 cells and (b) NIH3T3 cells. pDNA was pRL-CMV. The charge ratio of AF-liposomes/pDNA was 1.6. CyDs were added to AF-liposomes suspension before freeze-drying. The concentrations of *γ*-CyDs were 1 *μ*M/*μ*M lipids. Cells were incubated with pDNA/*γ*-CyDs/AF-liposomes for 3 h in FCS-free medium. After washing twice, the cells were incubated for 21 h in culture medium supplemented with 10% FCS. The luciferase activity in cell lysates was determined using a luminometer. Each value represents the mean ± SEM of 3–7 experiments. *P < .05  versus without CyD.

To evaluate the enhancing effects of *γ*-CyD on gene transfer activity of AF-liposomes associating pDNA encoding EGFP controlled by a CMV or SV40 promoter, we examined EGFP and firefly luciferase gene expression after transfection of pDNA/*γ*-CyDs/AF-liposomes in HepG2 cells (Figure 5). The extent of EGFP-expressing cells in the pDNA/AF-liposomes system without CyDs was found to be 8%, while that with *γ*-CyD and HP-*γ*-CyD were 19% and 10%, respectively (Figure 5(a)). Additionally, the enhancing effect of *γ*-CyD was observed in the pGL3-control vector encoding firefly luciferase and having a SV40 promoter (Figure 5(b)). These results suggest that the enhancing effect of *γ*-CyD on gene transfer activity of AF-liposomes is a gene- and promoter-independent manner. To confirm whether pDNA/*γ*-CyD/AF-liposomes have ASGP-R-mediated gene transfer activity, we performed transfection experiments in HepG2 cells in the presence and absence of AF, as an ASGP-R competitive inhibitor. Here, we confirmed that ASGP-R are expressed in HepG2 cells by the RT-PCR method (data not shown), which is consistent with previous findings [33–35]. As shown in Figure 6, gene transfer activity of AF-liposomes was markedly inhibited by the addition of AF, but not BSA, a control protein. These results suggest that AF-liposomes had the ASGP-R-mediated gene transfer activity. Interestingly, the similar enhancing effects of *γ*-CyD on Renilla luciferase protein expression after transfection of pDNA/AF-liposomes were, however, observed in A549 cells and NIH3T3 cells, ASGP-R negative cells (Figure 7). These results suggest that *γ*-CyD enhances the transfection efficiency of pDNA/AF-liposomes in ASGP-R-independent manner.

### 3.3. Cytotoxicity

**Figure 8:**
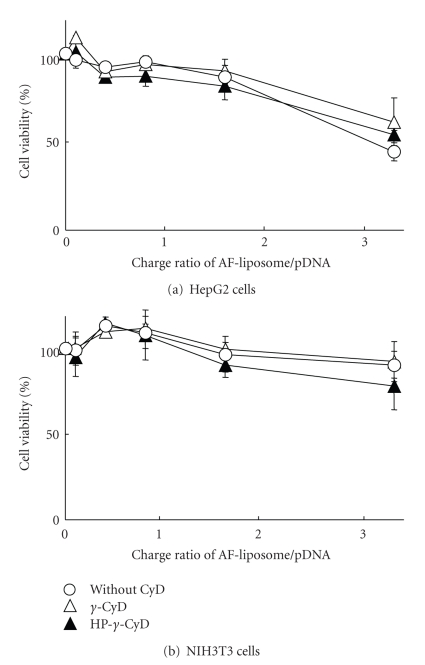
Cytotoxicity of pDNA/AF-liposomes or pDNA/*γ*-CyDs/AF-liposomes with various charge ratios in (a) HepG2 and (b) NIH3T3 cells. The concentrations of *γ*-CyDs were 1 *μ*M/*μ*M lipids. Cells were incubated with pDNA/*γ*-CyDs/AF-liposomes for 3 h in FCS-free medium. Cell viability was assayed by the WST-1 method. Each point represents the mean ± SEM of 3 experiments.

To reveal cytotoxicity of pDNA/CyDs/AF-liposomes, we examined the WST-1 method (Figure 8). Although the cell viability after treatment with pDNA/AF-liposomes and pDNA/CyDs/AF-liposomes for 3 h slightly decreased as a charge ratio of AF-liposomes/pDNA was increased in both HepG2 and NIH3T3 cells, that is, more than 80% of cell viability after application of pDNA/CyDs/AF-liposomes was observed at the charge ratio of 1.6 used in the transfection study as described above. These results suggest that pDNA/CyDs/AF-liposomes have great advantages as a nonviral vector, that is, superior transfection efficiency and less cytotoxicity, and the enhancing effect of *γ*-CyD on gene transfer activity of pDNA/AF-liposomes is not associated with cytotoxicity.

### 3.4. Effects of *γ*-CyD on Physicochemical Properties of pDNA/AF-Liposomes

To clarify physicochemical properties of the pDNA/AF-liposomes, we determined the particle sizes and *ζ*-potential values of the pDNA/*γ*-CyD/AF-liposomes at the charge ratio of 1.6. The mean diameter of the pDNA/*γ*-CyD/AF-liposomes was smaller than that of pDNA/AF-liposomes or pDNA/HP-*γ*-CyD/AF-liposomes (Table 2). Meanwhile, the *ζ*-potential values of pDNA/AF-liposomes, pDNA/*γ*-CyD/AF-liposomes, and pDNA/HP-*γ*-CyD/AF-liposomes were almost comparable (Table 2). These results indicate that *γ*-CyD reduced the particle size of AF-liposomes but did not change the *ζ*-potential value of pDNA/CyD/AF-liposomes.

 Next, we examined the effects of *γ*-CyDs on encapsulation ratios of pDNA into AF-liposomes (Table 3). The encapsulation ratio of pDNA in pDNA/*γ*-CyD/AF-liposomes was significantly higher than those of pDNA/HP-*γ*-CyD/AF-liposomes and pDNA/AF-liposomes. Meanwhile, the encapsulation ratios of *γ*-CyD and HP-*γ*-CyD into AF-liposomes were approximately 10.2% and 11.0%, respectively. These results suggest that *γ*-CyD improves the encapsulation of pDNA into AF-liposomes, although the extent of *γ*-CyD encapsulation into AF-liposomes is not high. In addition, both the encapsulation ratio of pDNA into AF-liposomes and gene transfer activity of pDNA/AF-liposomes were raised, as the number of the freeze-thaw cycle was increased, suggesting that the encapsulation of pDNA in AF-liposomes is correlated with the gene transfer activity of pDNA/AF-liposomes (Table 4).

### 3.5. Effects of *γ*-CyD on Membrane Fluidity of Liposomes

**Figure 9:**
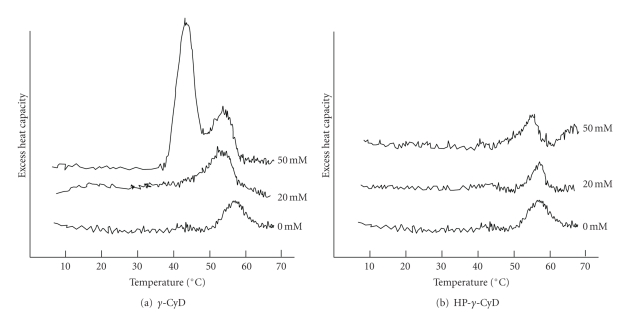
DSC thermograms of the gel-to-fluid state transition of N-liposomes at various concentrations of *γ*-CyDs. *γ*-CyDs were added to N-liposomes suspension. The concentration of total lipids of N-liposomes was 5 mM. The experiments were performed in the range of 4°C to 70°C using a Microcal MC2 apparatus. The temperature scanning rate was 1°C/min.

It is known that membrane fluidity of liposomes affects the release profiles as well as a retention time of drug encapsulated into liposomes. Therefore, we investigated the effects of *γ*-CyD on membrane fluidity of AF-liposomes using a Microcal MC2 scanning calorimeter. In the present study, we utilized N-liposomes to eliminate the effects of the heat degeneration of AF in a DSC thermograph. Figure 9 shows the effects of *γ*-CyD and HP-*γ*-CyD on DSC thermograms of N-liposomes (5 mM of total lipids). The peak derived from gel-to-fluid state transition in N-liposomes was observed at 55°C, while new peak was appeared at 42°C in the presence of 50 mM of *γ*-CyD (Figure 9(a)). On the other hand, no significant change in DSC thermographs was observed in the presence of HP-*γ*-CyD (Figure 9(b)). These results suggest that *γ*-CyD may affect the membrane fluidity of N-liposomes. 

**Figure 10:**
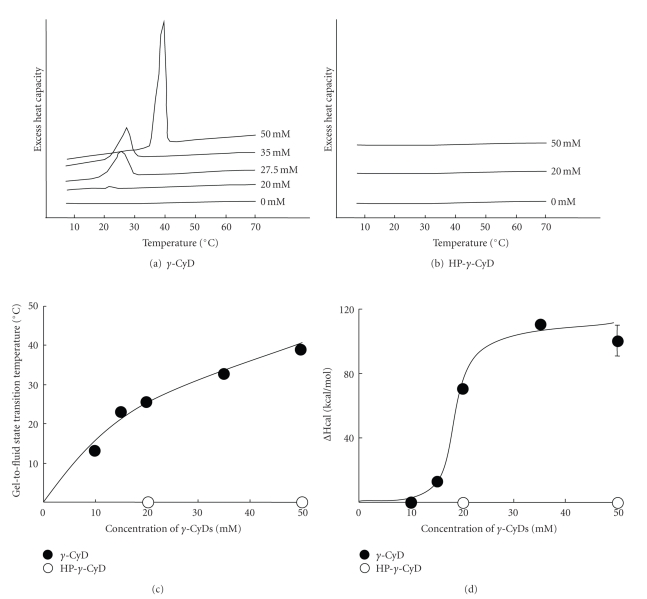
DSC thermograms (a, b), gel-to-fluid state transition temperature (c), and ΔHcal value (d) of the gel-to-fluid state transition of DLPC-liposomes at various concentrations of *γ*-CyDs. *γ*-CyDs were added to DLPC-liposomes suspension. The DLPC concentration was 5 mM. The experiments were performed in the range of 4°C to 70°C using a Microcal MC2 apparatus. The temperature scanning rate was 1°C/min. The gel-fluid state transition temperature and ΔHcal were taken at a peak temperature and a peak area of the DSC thermograms, respectively. Each point represents the mean  ±  SEM of 3 experiments.

 Generally, membrane fluidity and phase transition of liposomes are determined by the intensity of lipid-lipid interactions such as hydrophobic interaction, van der Waals forces, and hydrogen bond. Therefore, to evaluate the effects of *γ*-CyD on lipid-lipid interactions in AF-liposomes, we performed DSC analysis of DLPC-liposomes in the presence and absence of *γ*-CyD. The reason why we used DLPC-liposomes is due to clear observation of lipid-lipid interaction using DLPC composed of AF-liposomes. Figure 10 shows the effects of *γ*-CyD and HP-*γ*-CyD on DSC thermograms, phase transition temperature, and enthalpy (ΔHcal) of DLPC-liposomes (5 mM of total lipids). The phase transition temperature (Tc) of DLPC-liposomes in the presence of *γ*-CyD was shifted to high temperature as the concentration of *γ*-CyD was increased (Figures 10(a) and 10(c)). The ΔHcal value of DLPC-liposomes in the *γ*-CyD system was drastically elevated at 20 mM of *γ*-CyD (Figure 10(d)). Meanwhile, in the case of HP-*γ*-CyD, there was no significant change in DSC thermograms, phase transition temperature, and the ΔHcal values (Figures 10(b), 10(c) and 10(d)). Taken together, these results strongly suggest that *γ*-CyD enhances the lipid-lipid interaction of DLPC-liposomes, leading to the membrane stabilization of DLPC-liposomes.

### 3.6. Cellular Uptake of pDNA/AF-Liposomes

**Figure 11:**
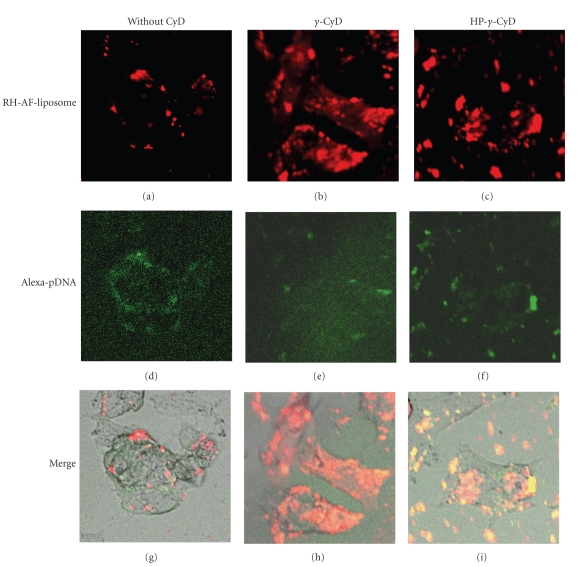
Confocal laser microscopic images for distribution of RH-AF-liposomes and Alexa-pDNA in HepG2 cells. Magnification: ×200. The charge ratio of AF-liposomes/pDNA was 1.6. *γ*-CyDs were added to AF-liposomes suspension before freeze-drying. The concentrations of *γ*-CyDs were 1 *μ*M/*μ*M lipids. Cells were incubated with Alexa-pDNA/*γ*-CyDs/RH-AF-liposomes for 3 h in FCS-free medium. After washing twice, the cells were observed using a confocal laser scanning microscopy.

Next, we examined the cellular uptake of pDNA/*γ*-CyD/AF-liposomes into HepG2 cells using a CLSM. Figure 11 shows the CLSM images for distribution of RH-AF-liposomes and Alexa-pDNA in HepG2 cells at 3 h after transfection. The strong fluorescence derived from RH-AF-liposomes and Alexa-pDNA in the presence of *γ*-CyD was mainly observed in cytoplasm of HepG2 cells. Meanwhile, the fluorescence RH-AF-liposomes and Alexa-pDNA in the absence of CyD and with HP-*γ*-CyD was mainly observed on cell surface. Hence, these results suggest that pDNA/*γ*-CyD/AF-liposomes can be internalized into HepG2 cells to a larger extent, compared to pDNA/AF-liposomes and pDNA/*γ*-CyD/AF-liposomes.

## 4. Discussion

In this study, we clarified that pDNA/*γ*-CyD/AF-liposomes have potent hepatocyte-selective gene transfer activity and negligible cytotoxicity, compared to pDNA/AF-liposomes and pDNA/HP-CyDs/AF-liposomes.

 In cationic liposome-mediated gene transfection, lipid composition and lipid type are the most important physicochemical factors, because they affect not only the interaction with pDNA but also the affinity to target cells [36, 37]. In the present study, we prepared AF-liposomes with a lipid composition of TMAG/DOPE/DLPC/DPPE (2/4/3/1, molar ratio). DOPE is known to enhance the endosomal escape of pDNA due to its structural change into hexagonal II form at pH 5-6, an endosomal pH range, resulting in destabilizing endosomal membranes [38, 39]. TMAG, a cationic lipid, makes it possible to interact with pDNA in AF-liposomes. Additionally, DPPE was used as a binding lipid with AF. Actually, cationic liposomes composed of TMAG/DOPE/DLPC (1/2/2, molar ratio) are commercially available transfection reagents as GeneTransfer, which have already been utilized in a clinical trial for a nonviral vector to deliver the interferon-*β* gene for the treatment of brain tumor in Japan [40]. Therefore, we used AF-liposomes composed of these lipids in the present study. 

The most important finding found in the present study is that *γ*-CyD enhances transfection efficiency of pDNA/AF-liposomes in HepG2 cells (Figures 3–5) with negligible cytotoxicity (Figure 8). The enhancing mechanisms of *γ*-CyD presumed are discussed as follows. 

In the present study, we revealed that transfection efficiency of the pDNA/*γ*-CyD/AF-liposomes, not N-liposomes, was inhibited by the addition of AF in HepG2 cells (Figure 6). Meanwhile, in NIH3T3 cells, transfection efficiency of the pDNA/AF-liposomes was not suppressed by the addition of AF. These results strongly suggest that pDNA/*γ*-CyD/AF-liposomes can be entered HepG2 cells through ASGP-R-mediated endocytosis, consistent with Aramaki and his colleague's report [41], and the enhancing effect of the *γ*-CyD may by associated with the ASGP-R-mediated endocytosis. However, *γ*-CyD also enhanced gene transfer activity of pDNA/AF-liposomes even in A549 cells and NIH3T3 cells, ASGP-R negative cells (Figure 7). These results suggest that the enhancing effect of *γ*-CyD on transfection efficiency of pDNA/AF-liposomes is in an ASGP-R-independent manner. 

 The particle sizes and encapsulation ratio of pDNA/*γ*-CyD/AF-liposomes should be involved in the enhancing effect of *γ*-CyD on gene transfer activity of AF-liposomes. The particle size of pDNA/*γ*-CyD/AF-liposomes was decreased in the presence of *γ*-CyD, although the *ζ*-potential value of pDNA/*γ*-CyD/AF-liposomes was almost equivalent to that of pDNA/AF-liposomes and pDNA/HP-*γ*-CyD/AF-liposomes (Table 2), suggesting that *γ*-CyD inhibits the aggregation of pDNA/AF-liposomes, because the particle size shows more than 200 nm, despite the fact that the liposomes were extruded through a filter membrane having a pore size of 200 nm. Meanwhile, the encapsulation ratio of pDNA was significantly increased by adding *γ*-CyD to pDNA/AF-liposomes (Table 3). Thus, these lines of evidence speculate that addition of *γ*-CyD enhances cellular uptake of pDNA/AF-liposomes. In fact, the CLSM study demonstrated that cellular uptake of pDNA/*γ*-CyD/AF-liposomes was higher than that of pDNA/*γ*-CyD/AF-liposomes (Figure 11). Furthermore, we confirmed that transfection efficiency of pDNA/*γ*-CyD/AF-liposomes was increased, as the encapsulation ratio of pDNA into pDNA/*γ*-CyD/AF-liposomes was augmented (Table 4). In view of the findings, the particle size of pDNA/*γ*-CyD/AF-liposomes and encapsulation ratio of pDNA into pDNA/*γ*-CyD/AF-liposomes are crucial role for enhancing transfection efficiency of pDNA/AF-liposomes. 

 The important question regarding the enhancing effect of *γ*-CyD on transfection efficiency of pDNA/AF-liposomes still remains, because three types of HP-CyDs did not have the enhancing effect. To address this question, the DSC analysis was performed. This study indicated that *γ*-CyD, but not HP-*γ*-CyD, changed membrane fluidity and stabilized the N-liposomal membranes (Figure 9). In fact, *γ*-CyD increased the Tc value of DLPC-liposomes, although HP-*γ*-CyD did not increase anymore (Figure 10). This increase in the Tc value induced by *γ*-CyD could be attributed to a compactness of lipid bilayer of DLPC liposomes. It is thereby possible that *γ*-CyD may increase Tc values of the other liposomes such as DMPC, DPPC, and DSPC. Here, it is well known that *γ*-CyD is highly hydrophilic and surface inactive [17]. Therefore, we presumed that *γ*-CyD encapsulates the aqueous compartment of liposomes rather than in the bilayer of liposomes. Anyhow, it is clear that the magnification of the interaction of liposomal membranes containing DLPC with HP-*γ*-CyD is weaker than that with *γ*-CyD, possibly due to the steric hindrance of the HP group in a HP-*γ*-CyD molecule. Taken together, it is likely that the stabilizing effects of *γ*-CyD on AF-liposomal membrane may lead to inhibition of pDNA leakage from AF-liposomes and increase in cellular uptake of pDNA, eventually leading to the enhancement of *in vitro* transfection efficiency of pDNA/*γ*-CyD/AF-liposomes in cells. 

 Finally, we investigated the role of free *γ*-CyD on transfection efficiency of pDNA/*γ*-CyD/AF-liposomes in HepG2 cells. The physical mixture of pDNA/AF-liposomes and *γ*-CyD in culture medium had no enhancing effect on transfection efficiency of pDNA/AF-liposomes (data not shown). Therefore, encapsulation of *γ*-CyD into AF-liposomes may be pivotal for enhancing gene transfer activity. To reveal the detailed mechanism for the enhancing effect of *γ*-CyD associated in AF-liposomes on transfection efficiency of pDNA/AF-liposomes, further elaborate study should be necessary.

## 5. Conclusions

In the present study, we demonstrated that *γ*-CyD enhanced gene transfer activity of pDNA/AF-liposomes in not only HepG2 cells but also A549 and NIH3T3 cells, probably due to various effects of *γ*-CyD on AF-liposomes such as inhibition of aggregation of the liposomes, high encapsulation of pDNA into the liposomes, and stabilization of lipid bilayer of the liposomes. Consequently, the potential use of *γ*-CyD could be expected as an enhancer of gene transfer activity of AF-liposomes. Also, these data may be useful for design of cell-specific cationic liposomes as a nonviral vector.

## Figures and Tables

**Table 1:** Chemical structures of CyDs used in this study.

CyDs	*n*	R	D.S.^(a)^
*α*-CyD	1	H	
*γ*-CyD	3	H	
HP-*α*-CyD^(b)^	1	H or CH_2_CH(CH_3_)OH	4.0
HP-*β*-CyD^(c)^	2	H or CH_2_CH(CH_3_)OH	4.8
HP-*γ*-CyD^(d)^	3	H or CH_2_CH(CH_3_)OH	4.3
DM-*β*-CyD^(e)^	2	H or CH_3_	14

(a) The average degree of substitution, (b) 2-Hydroxypropyl-*α*-CyD, (c) 2-Hydroxypropyl-*β*-CyD, (d) 2-Hydroxypropyl-*γ*-CyD, and (e) 2, 6-Di-*O*-methyl-*β*-CyD.

**Table 2:** *ζ*-potential value and particle size of pDNA/AF-liposomes or pDNA/*γ*-CyDs/AF-liposomes.

CyD	Particle size	*ζ*-potential
	(nm)	(mV)
Without CyD	357.9 ± 17.7	21.5 ± 2.9
*γ*-CyD	277.9 ± 10.5*	17.0 ± 2.7
HP-*γ*-CyD	348.0 ± 16.5	22.3 ± 3.3

The charge ratio of AF-liposome/pDNA was 1.6. *γ*-CyDs were added to AF-liposome suspension before freeze-drying. The concentrations of *γ*-CyDs were 1 *μ*M/*μ*M lipids. The *ζ*-potential was measured by a light-scattering method. The particle size was determined using a photon correlation spectroscopic analyzer. Each value represents the mean ± SEM of 3 experiments. *P < .05  versus without CyD.

**Table 3:** Encapsulation ratios of pDNA and *γ*-CyDs into AF-liposomes.

CyD	Encapsulation ratio of pDNA (%)	Encapsulation ratio of *γ*-CyDs (%)
Without CyD	42.4 ± 4.3	—
*γ*-CyD	58.2 ± 1.8*	10.2 ± 0.5
HP-*γ*-CyD	41.9 ± 3.3	11.0 ± 2.2

The charge ratio of AF-liposome/pDNA was 1.6. *γ*-CyDs were added to AF-liposome suspension before freeze-drying. The concentrations of *γ*-CyDs were 1 *μ*M/*μ*M lipids. The encapsulation ratios of pDNA were determined using Picogreen assay. The encapsulation ratios of *γ*-CyDs were determined by an anthrone-sulfuric acid method. Each value represents the mean ± SEM of 3 experiments. *P < .05  versus without CyD.

**Table 4:** Encapsulation ratio of pDNA into AF-liposomes and gene transfer activity of pDNA/AF-liposomes at various cycles of freeze-thaw in HepG2 cells.

Freeze thaw cycle	Encapsulation ratio of pDNA (%)	Gene transfer activity (fold)
0	35.2 ± 0.9	0.56 ± 0.07
1	36.2 ± 0.5	0.59 ± 0.06
2	37.4 ± 2.1	0.76 ± 0.02
3	42.4 ± 4.3	1
5	44.1 ± 1.3	1.03 ± 0.02

The charge ratio of AF-liposomes/pDNA was 1.6. pDNA/AF-liposome was prepared using a freeze-thaw method which was repeated from 0 to 5 cycles. Cells were incubated with pDNA/*γ*-CyDs/AF-liposome for 3 h in FCS-free medium. After washing twice, the cells were incubated for 21 h in culture medium supplemented with 10% FCS. The luciferase activity in cell lysates was determined using a luminometer. Each value represents the mean ± SEM of 3 experiments.
